# Zearalenone and ß-Zearalenol But Not Their Glucosides Inhibit Heat Shock Protein 90 ATPase Activity

**DOI:** 10.3389/fphar.2019.01160

**Published:** 2019-10-18

**Authors:** Juan Antonio Torres Acosta, Herbert Michlmayr, Mehrdad Shams, Wolfgang Schweiger, Gerlinde Wiesenberger, Rudolf Mitterbauer, Ulrike Werner, David Merz, Marie-Theres Hauser, Christian Hametner, Elisabeth Varga, Rudolf Krska, Franz Berthiller, Gerhard Adam

**Affiliations:** ^1^Department of Applied Genetics and Cell Biology, University of Natural Resources and Life Sciences (BOKU), Vienna, Austria; ^2^Institute of Bioanalytics and Agro-Metabolomics, Department of Agrobiotechnology IFA-Tulln, University of Natural Resources and Life Sciences, Vienna (BOKU), Austria; ^3^Institute of Applied Synthetic Chemistry, Vienna University of Technology, Vienna, Austria; ^4^Institute for Global Food Security, School of Biological Sciences, Queens University Belfast, University Road, Belfast, United Kingdom

**Keywords:** *Arabidopsis*, HSP90, wheat, glycosylation, *Fusarium*, radicicol

## Abstract

The mycotoxin zearalenone (ZEN) is produced by many plant pathogenic *Fusarium* species. It is well known for its estrogenic activity in humans and animals, but whether ZEN has a role in plant–pathogen interaction and which process it is targeting *in planta* was so far unclear. We found that treatment of *Arabidopsis thaliana* seedlings with ZEN induced transcription of the *AtHSP90.1* gene. This heat shock protein (HSP) plays an important role in plant–pathogen interaction, assisting in stability and functionality of various disease resistance gene products. Inhibition of HSP90 ATPase activity impairs functionality. Because HSP90 inhibitors are known to induce *HSP90* gene expression and due to the structural similarity with the known HSP90 inhibitor radicicol (RAD), we tested whether ZEN and its phase I metabolites α- and ß-zearalenol are also HSP90 ATPase inhibitors. Indeed, *At*HSP90.1 and wheat *Ta*HSP90-2 were inhibited by ZEN and ß-zearalenol, while α-zearalenol was almost inactive. Plants can efficiently glycosylate ZEN and α/ß-zearalenol. We therefore tested whether glucosylation has an effect on the inhibitory activity of these metabolites. Expression of the *A. thaliana* glucosyltransferase UGT73C6 conferred RAD resistance to a sensitive yeast strain. Glucosylation of RAD, ZEN, and α/ß-zearalenol abolished the *in vitro* inhibitory activity with recombinant HSP90 purified from *Escherichia coli*. In conclusion, the mycotoxin ZEN has a very prominent target in plants, HSP90, but it can be inactivated by glycosylation. This may explain why there is little evidence for a virulence function of ZEN in host plants.

## Introduction

The mycotoxin zearalenone (ZEN) has potent estrogenic activity in humans and animals (Kuiper-Goodman et al., 1987; Zinedine et al., 2007), and based on risk assessments, it is a regulated mycotoxin in food in Europe (European Commission, 2006b; Gromadzka et al., 2008; EFSA Panel on Contaminants in the Food Chain (CONTAM), 2011). In addition, guidance levels for feed exist which reflect differences in metabolization of ZEN in different animal species (European Commission, 2006a; European Commission, 2016). ZEN is produced by multiple species of *Fusarium*, particularly by *Fusarium graminearum* in the broad sense, which has been split into 16 species based on molecular phylogeny (Sarver et al., 2011; van der Lee et al., 2015). Also *Fusarium culmorum*, *Fusarium crookwellense* (synonym *Fusarium* *cerealis*), *Fusarium equiseti*, *Fusarium pseudograminearum*, and *Fusarium semitectum* consistently produce ZEN (Desjardins, 2006), as do more recently described species, such as *Fusarium praegraminearum* (Grafenhan et al., 2016) and *Fusarium dactylidis* (Aoki et al., 2015). Sporadic production has also been reported for other species (e.g., isolates of *Fusarium solani* (Richardson et al., 1985), *Fusarium sporotrichioides* (Molto et al., 1997), *Fusarium oxysporum* (Milano and Lopez, 1991), *Fusarium tricinctum* (Caldwell et al., 1970), and *Fusarium heterosporum* (Bottalico et al., 1989)), but this may be at least partly due to errors in identification solely based on microscopy. Nevertheless, ZEN production is widespread, particularly in pathogens of small grain cereals and corn, which suggests it might have a role as a virulence factor. Estrogen receptor signaling is absent in plants, and the biological mode of action of ZEN *in planta* remained so far unclear. Kim et al. (2005) reported unchanged virulence of ZEN-deficient knockout mutants on infected barley heads. Also, Gaffoor and Trail (2006) reported unaltered virulence after point inoculation of wheat heads. Likewise, Lysoe et al. (2006) observed no difference in a root rot assay with barley seedlings dipped into spore suspensions. However, gene expression studies revealed that the ZEN biosynthesis genes were not expressed under the conditions used to infect wheat and barley (Lysoe et al., 2011), and similar results have been obtained for corn (Harris et al., 2016). Concluding that ZEN plays no role in virulence might therefore be premature.

**Figure 1:**
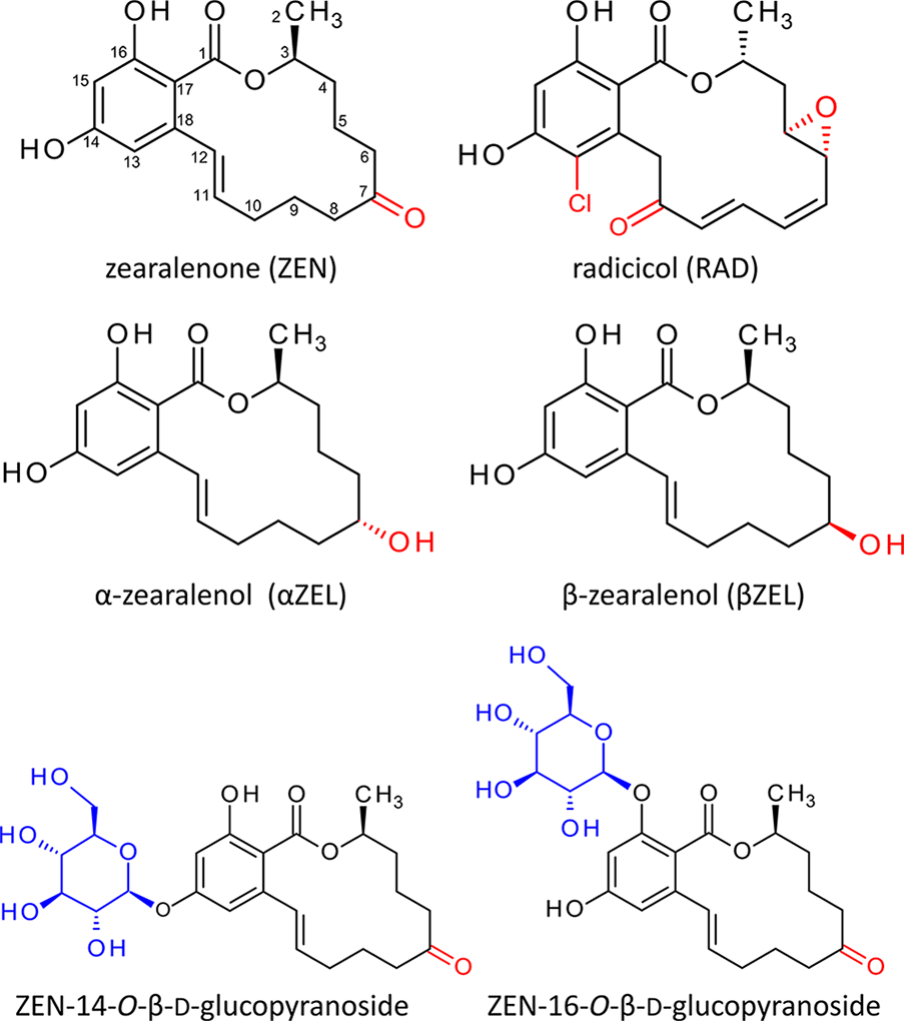
Structures of radicicol, zearalenone (Metzler, 2011), and the zearalenone phase I metabolites α- and ß-zearalenol. Structural differences are highlighted red. Below the structures of zearalenone-14-*O*-ß-D-glucopyranoside and zearalenone-16-*O*-ß-D-glucopyranoside. The glucosides of α-zearalenol and ß-zearalenol produced in this study carry the glucose moiety at the analogous position.

The structure of ZEN (**Figure 1**) is similar to that of radicicol (RAD); both are members of the class of resorcylic acid lactones (Mirrington et al., 1965; Metzler, 2011). RAD (also known as monorden) was originally isolated from *Nectria radicicola*. RAD strongly affects expression and inhibits the activity of heat shock proteins (HSPs), particularly of the HSP90 family (Schulte et al., 1998; Roe et al., 1999; Yamada et al., 2007). Products of the *HSP90* gene family play a crucial role in cellular processes such as protein folding, maturation, activation, transport, and degradation. Although most cellular proteins do not require HSP90 for folding under normal conditions, important client proteins such as transcription factors or signal transduction components (Picard, 2002) do, and consequently, HSP90 activity is essential for life.

Binding and subsequent hydrolysis of ATP is the key mechanism through which HSP90 members interact with their client proteins. Inhibition of HSP90 ATPase activity has been thoroughly studied, and the nucleotide (ATP) binding domain (histidine kinase like ATPase superfamily, pfam02518) is highly conserved within the HSP90 family. Therefore, drugs intended to reduce HSP90 functionality, for example, in cancer therapy (Roe et al., 1999; Sgobba and Rastelli, 2009), mainly target the ATP binding site. It has been shown that RAD and the *Streptomyces* metabolite geldanamycin target the same *N*-terminal ATP binding site and strongly inhibit the ATPase activity of HSP90 members (Roe et al., 1999; Rowlands et al., 2004).

Based on results of *Arabidopsis thaliana* microarray data after ZEN treatment and the structural similarity between ZEN and RAD, we hypothesized that ZEN may also be an HSP90 inhibitor. *In planta*, the ZEN phase I plant metabolites α- and β-zearalenol (α/βZEL) and phase II glucoconjugates (Berthiller et al., 2006) are formed (**Figure 1**). The 14-*O*-β-D-glucopyranosides of ZEN (ZEN14G) and α/βZEL have previously been described to occur in different plant species (Berthiller et al., 2006; Berthiller et al., 2009a; Berthiller et al., 2009b). More recently, ZEN-16-*O*-β-D-glucopyranoside (ZEN16G) (**Figure 1**) has been found in ZEN-treated barley, wheat, and *Brachypodium* (Kovalsky Paris et al., 2014) but also in naturally infected grain samples (Nathanail et al., 2015).

Here, we tested whether ZEN and its phase I detoxification derivatives (α/βZEL) are HSP90 ATPase inhibitors and, furthermore, whether glycosylation has an effect on the HSP90 ATPase inhibitor activity.

## Materials and Methods

### Chemicals and Materials

In the phosphate assay, absorbance at 620 nm was determined using a PerkinElmer EnSpire 2300 Multilabel Plate Reader. F-bottom 96-well cell culture plates were obtained from Greiner Bio-One (Kremsmuenster, Austria). Malachite green, polyvinyl alcohol, ammonium molybdate, ATP sodium salt, RAD, α- and ßZEL, as well as Murashige and Skoog basal medium were purchased from Sigma-Aldrich (Vienna, Austria). In order to avoid contamination with inorganic phosphate, glassware and pH meter electrodes were rinsed extensively with double-distilled water before use.

ZEN14G, the 14-*O*-glucosides of α- and ßZEL, and ZEN16G were synthesized as previously reported (Poppenberger et al., 2006; Krenn et al., 2007; Berthiller et al., 2009a; Kovalsky Paris et al., 2014).

Analogously, the 16-*O*-glucosides of α- and ßZEL have been prepared by chemical reduction of ZEN16G with sodium borohydrate and subsequent high-performance liquid chromatography (HPLC) preparation of the mixture of αZEL-16-*O*-glucoside and ßZEL-16-*O*-glucoside (Michlmayr et al., 2017). Enhanced product ion (EPI) tandem mass spectrometry (MS/MS) scans to confirm the identity of all used glucosides were acquired on a 4000 QTrap mass spectrometer (Sciex, Framingham, USA), coupled to a 1290 UHPLC system (Agilent, Waldbronn, Germany) after negative electrospray ionization. Chromatograms and MS/MS spectra for all used ZEN metabolites can be found in the supplementary material (**Supplementary Figures S1**–**S6**).

### Preparation of *Arabidopsis thaliana* Seedlings

*A. thaliana* (ecotype Columbia, Col-0) seeds were surface-sterilized using sodium hypochlorite (2%) plus 0.01% Triton X for 10 min and rinsed twice with sterile water. Seeds were distributed in 24-well plates (4 wells per treatment, 30–40 seeds per well) and re-suspended in 1 ml (per well) of liquid Murashige and Skoog medium (“MS” basal salt mixture, Sigma) supplemented with 1% sucrose. After 2 days of stratification at 4°C, the plates were transferred to a growth chamber at 21°C under a 16 h light/8 h dark photoperiod.

### Microarray Data

The response of the *A. thaliana* transcriptome to treatment with 50 µM ZEN after 2 and 24 h had been previously determined (Werner, 2005) using ATH1 22K Affymetrix gene chips at the NASC’s International Affymetrix Service (http://affymetrix.arabidopsis.info/), and the results and experimental conditions were deposited in the NASC International Affymetrix Service database as NASCARRAYS-71 and the EMBL-EBI ArrayExpress database as E-NASC-52 (http://www.ebi.ac.uk/arrayexpress/experiments/E-NASC-52/). Treatments in this experiment were with 50 µM ZEN for 2 and 24 h. As control, seedlings were treated with the same concentration of solvent (dimethyl sulfoxide, DMSO).

### Expression Analysis of *ATHSP90.1* by Quantitative Real-Time PCR

Total RNA was extracted from 13-day-old *A. thaliana* (Col-0) seedlings grown in liquid MS medium supplemented with 2.5% sucrose and 50 μM ZEN for 2 h. Samples were snap-frozen and stored at −80°C. RNA was extracted with TRI reagent TR (Molecular Research Center, Inc. Cincinnati, Ohio, USA). As control, seedlings were treated with the same concentration of solvent (DMSO). RNA was quantified with the Qubit (Invitrogen) and NanoDrop (Peqlab) systems. First-strand cDNA was synthesized from 2.5 μg RNA after RNAse free DNase I digestion (Fermentas) with Superscript III reverse transcriptase (Invitrogen) as described in Karsai et al. (2002). RT-qPCR expression analysis was performed using the Hot FirePol EvaGreen qPCR Mastermix (Solis Biodyne) with a Rotorgene 3000 (Qiagen). Primers for *AtHSP90.1* were HSP90.1-RTfw 5’-ctctcacgagtgggaactcatc-3’ and *A*tHSP90-1-A 5’-TTGAATTCTAGCTGACCCTCC-3’ and the reference gene *ADAPTOR PROTEIN-2* *MU-ADAPTIN* (*AP2M; AT5G46630*) AP2M_F 5’-CAATCGATTGCTTGGTTTGGA-3’ and AP2M_R 5’-CGAACTCGCAGACCAGATGC-3’. Absolute and relative expression were calculated with a dilution series of purified *AtHSP90.1* and *AP2M* PCR fragments of known molar concentration in each RT-qPCR run. The amplicon sizes of *AtHSP90.1* and *AP2M* were 164 and 171 bp, respectively. The PCR efficiencies of *AtHSP90.1* and *AP2M* were 1.01 and 0.93, respectively. Each sample was measured in triplicate from four independent cDNAs. Amplicon identity was verified by melting curve analysis.

### Cloning, Expression, and Purification of HSP90 Proteins

For initial experiments, we expressed and purified the *Saccharomyces cerevisiae* *Sc*Hsp82 protein. The *HSP82* coding region was amplified using the primer pair HSP82-1fwd (5’-CGCGGATCCATGGCTAGTGAAACTTTTG-3’) and HSP82-2rev (5’-GCGCGAAGCTTCTAATCTACCTCTTCCATT-3’), which contain *Bam*HI and *Hin*dIII sites (underlined), respectively. The resulting 2,156 bp fragment was digested with *Bam*HI and *Hin*dIII and ligated into the expression vector pQE-80L (Qiagen) cleaved with the same enzymes yielding plasmid pGW870. The coding region of *AtHSP90.1* (*At5g52640*) was amplified from cDNA with the oligonucleotide primers 5’-CCCCATATGGCGGATGTTCAGATGGC-3’ and 5’-GGACTAGTGTCGACTTCCTCCATCTTGCTC-3’. The resulting PCR product was digested with *Nde*I and *Spe*I (restriction sites on primers underlined) and introduced to pQE-80L (Qiagen), cleaved with the same enzymes to obtain the construct pQE80:*At*HSP90.1. The cDNA of wheat *HSP90* gene *TaHSP90-2* (*Triticum aestivum*; DQ665784.1) was amplified with 5’-CCCCATATGGCGACGGAGACCGAGACC-3’ and 5’-GGACTAGTGTCGACCTCCTCCATCTTGC-3’ (*Nde*I and *Spe*I, respectively) and introduced to pQE-80L. All constructs were verified by sequencing.

For production of 6xHis-tagged fusion proteins, the plasmids were introduced into either the standard expression host BL21 (DE3) or the phosphatase-deficient *Escherichia coli* strain K894 (*garB10* *fhuA22 phoA4*(Am) *phoR79::Tn10* *ompF627(T2R) serU132(AS) fadL701(T2R) relA1 pitA10 spoT1 rrnB-2 mcrB1 creC510*) obtained from the Yale *E. coli* stock center (#7785).

Protein production was induced by isopropyl β-D-1-thiogalactopyranoside (IPTG) following the supplier’s instructions. Protein purification was performed on an Äkta system (GE Healthcare, Uppsala, Sweden). The recombinant proteins were purified by immobilized metal affinity chromatography on Ni^2+^ charged Chelating Sepharose (GE Healthcare) and eluted with 150 mM of imidazole. Further purification was done by anion exchange chromatography on a 1 ml Resource Q column (GE Healthcare). Proteins were bound to the column in 25 mM Tris/Cl pH 7.5 and eluted with a linear gradient of 0–1 M NaCl in 15 column volumes. The obtained fractions were desalted using a Sephadex™ G-25 fine column, and the purified recombinant proteins were stored in the final elution buffer (25 mM Tris/Cl pH 7.5) at 4°C.

### ATPase Activity Assay

The ATPase activities of recombinant proteins *At*HSP90.1, *Ta*HSP90-2, and *Sc*Hsp82 were assayed following the method of Rowlands et al. (2004) using an ATP concentration of 1 mM. The effect of several potential inhibitors was tested in 96-well plates using toxin concentrations ranging from 1 to 150 µM. All compounds were dissolved in 20% DMSO and further diluted. The assays were performed in a total volume of 25 µl with a final protein concentration of 0.2 µg/µl and 4% DMSO. Controls were incubated with the solvent DMSO without inhibitor. The reactions were incubated at 37°C for 3 h in the dark. After this time, the released inorganic phosphate was quantified with the malachite green colorimetric assay (Rowlands et al., 2004). The reaction was stopped by adding 10 µl of 34% sodium citrate to each well after 20 min incubation at room temperature, and the absorbance was measured at 620 nm.

### Production of RAD and RAD-Glucoside

For the production of RAD, baby food jars were filled with 10 g “Langkorn” rice and 10 ml of NaCl solution (100 mg NaCl per jar) and then autoclaved. For inoculation, 1 ml suspension containing about 3 × 10^6^ spores of *N. radicicola* (strain MA3441 of the Austrian Center of Biological Resources, http://acbr-database.boku.ac.at/) was spread on the rice, and the cultures were incubated at 20°C in the dark for 2 weeks. Then 25 ml of ethanol was added, and after homogenization with an IKA Ultra-Turrax, the extract (containing up to 2.7 g/L RAD) was filtered through Whatman® paper filters. The solvent was partially removed using a rotary evaporator (Buchi R210 Rotavap) at 38°C. To 100 ml of concentrated sample, 25 ml of ethyl acetate (EtOAc, J.T. Baker) was added, and after liquid–liquid partition, the organic phase was concentrated again. The solution was heated to approximately 60°C and slowly cooled down over a period of 3 days in the cold room to crystallize RAD. The residual solvent was removed by vacuum filtration and the crystals washed three times with Milli-Q ultrapure water (Millipore, Molsheim, France). For further purification, preparative HPLC (1100 series, Agilent Technologies, Waldbronn, Germany) was applied. For HPLC separation, a semi-preparative reversed-phase column, Phenomenex Gemini C18, 250 × 10 mm, 5 µm; flow rate: 16 ml/min; eluent A: 90:10 (v:v) Milli-Q H_2_O:methanol (MeOH) and eluent B: 100% MeOH (LC grade, Merck, Darmstadt, Germany); and injection volume of 500 µl were used. The column was equilibrated with 10% MeOH, which was held for 1 min. A linear gradient was run from 10% to 100% MeOH within 10 min and a hold time of 3 min.

For RAD-glucoside preparation, the genetically modified yeast strain YGZA515 transformed with plasmid pBP918 (Poppenberger et al., 2006), leading to expression of *At*UGT73C6, was employed. First, 30 L of log phase cells (OD_600_ 1.1) was produced in synthetic complete medium without leucine (SC-LEU) medium in a bioreactor (Applikon, pH 4, 30°C, 100% oxygen saturation, 10 L/oxygen per hour, and stirring at 600 rpm). The yeast was harvested by centrifugation and the cell paste was transferred into 5 L of fresh SC-LEU medium in a small batch fermenter (pH 5, 30°C, 4.5 g/L glucose, 10 L/oxygen per hour, stirring at 400 rpm). A short heat shock (37°C) was applied prior to RAD addition to induce the expression of stress response genes. A total amount of 18.2 mg RAD was fed to the yeast culture in three equal portions in 1 h intervals. After 46 h, the cells were pelleted by centrifugation, and the supernatant was extracted with an equal amount of EtOAc. The organic phases were collected, transferred into a 1,000 ml rotating flask, and evaporated using a rotary evaporator (Buchi R210 Rotavap) at 40°C. The residue was taken up in 50 ml MeOH and filtrated. The concentrated extract was cleaned of impurities by column chromatography on silica gel (Merck®) using a glass tube with a diameter of 30 mm and a height of 50 cm (silica gel bed 20 cm) using 100% EtOAc to 100% MeOH as mobile phases. Fractions containing RAD-glucoside were concentrated and selected for further purification. Preparative HPLC (1100 series, Agilent Technologies, Waldbronn, Germany) was employed using a semi-preparative column (Phenomenex Gemini C18, 250 × 10 mm, 5 µm). The column temperature was held isothermally at 25°C, and the flow rate was kept constant at 16 ml/min. The injection volume was 500 µl, and the mobile phases were A: MeOH:H_2_O (20:80, v:v) and B: 100% MeOH LC grade (Merck, Darmstadt, Germany). The chromatography was achieved using binary gradient elution and initiated with MeOH:H_2_O (20:80) for 2 min, and thereafter, the MeOH content was increased to 100% over 12 min, a hold time of 4 min at the same composition, and then back to the initial conditions over a period of 4 min. RAD-glucoside could be detected by its UV signal at 264 nm. RAD-glucoside–containing fractions were pooled, the solvents were evaporated, and the residue transferred into an 8 ml dark glass vial, evaporated to dryness under N_2_ and weighed (4.78 mg) using a microbalance (MECAPLEX Switzerland). The purity of RAD-glucoside was tested using an Agilent 1100 HPLC system with a DAD detector [mobile phase aqueous ACN 0%→100%, UV at 200 nm, column: Phenomenex Gemini C18 (150 × 4.6 mm, 5 µm)]. The sum formula of the purified compound was verified by high-resolution mass spectrometry (HR-MS) on an LTQ Orbitrap XL mass spectrometer (Thermo Fisher Scientific, Waltham, MA, USA) after negative electrospray ionization.

## Results

### ZEN Induces the Expression of *ATHSP90.1*

**Figure 2:**
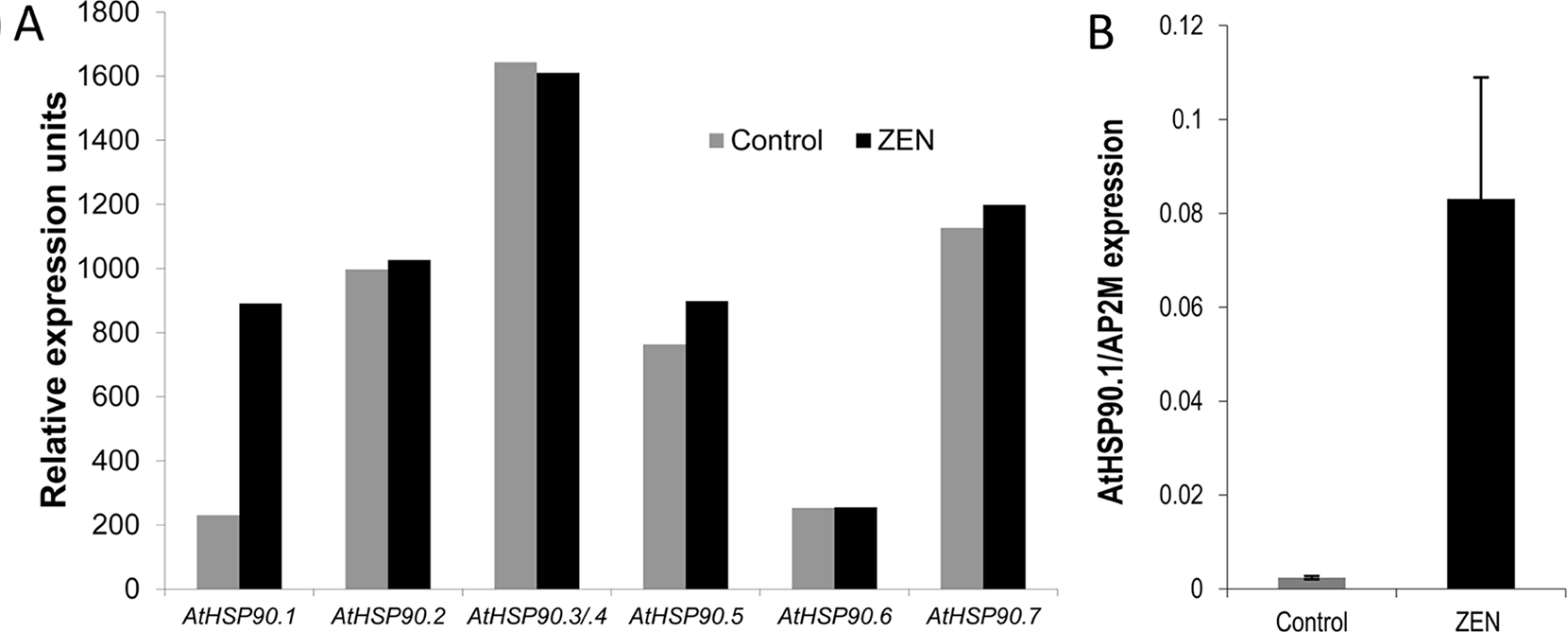
Transcriptional response of *Arabidopsis thaliana* *HSP*90 genes to zearalenone (ZEN). **(A)** Expression levels of *A. thaliana HSP*90 genes after 2 h treatment with ZEN (50 µM) or solvent (control; dimethyl sulfoxide, DMSO) according to microarray data from Werner (2005). The genes *HSP90.3* and *HSP90.4* cannot be distinguished by the probes on the chip (labeled *HSP90.3/4*). **(B)** Quantitative real-time PCR of *A. thaliana* *HSP90.1* after 2 h treatment with ZEN (50 µM) or DMSO. The results are expressed as relative expression to the reference gene *ADAPTOR PROTEIN-2* *MU-ADAPTIN* (*AP2M; AT5G46630*) and represent the means of four biological replicates ± standard deviation.

The transcriptional responses of *A. thaliana* after 2 and 24 h treatments with 50 µM ZEN (corresponding to 15.9 mg/L) were previously studied (Werner, 2005). Besides putative detoxification enzymes (e.g., ABC transporters and UDP-glucosyltransferases), several types of (small) HSPs were found upregulated in this study. In *A. thaliana*, the *HSP90* family consists of seven genes: the encoded proteins are located in the cytosol (*A*tHSP90.1 to *At*HSP90.4), in chloroplasts (*At*HSP90.5), in mitochondria (*At*HSP90.6), or in the endoplasmic reticulum (*At*HSP90.7) (Krishna and Gloor, 2001). *At*HSP90.1 is highly stress inducible and of particular importance for disease resistance (Takahashi et al., 2003). It has previously been reported that treatment of *A. thaliana* seedlings with RAD induced the *AtHSP90.1* promoter (Yamada et al., 2007). According to the microarray results, *AtHSP*90.1 was induced about 3.7-fold after 2 h, while the other members were seemingly unaffected (**Figure 2A**). Using the stable housekeeping gene AT5G46630 as a reference (Wang et al., 2014), quantitative real-time PCR revealed a 35-fold (t-test, *p* = 0.00372) induction of *AtHSP90.1* by ZEN in comparison to the mock control DMSO (**Figure 2B**).

### ZEN and βZEL Inhibit ATPase Activity of HSP90

**Figure 3:**
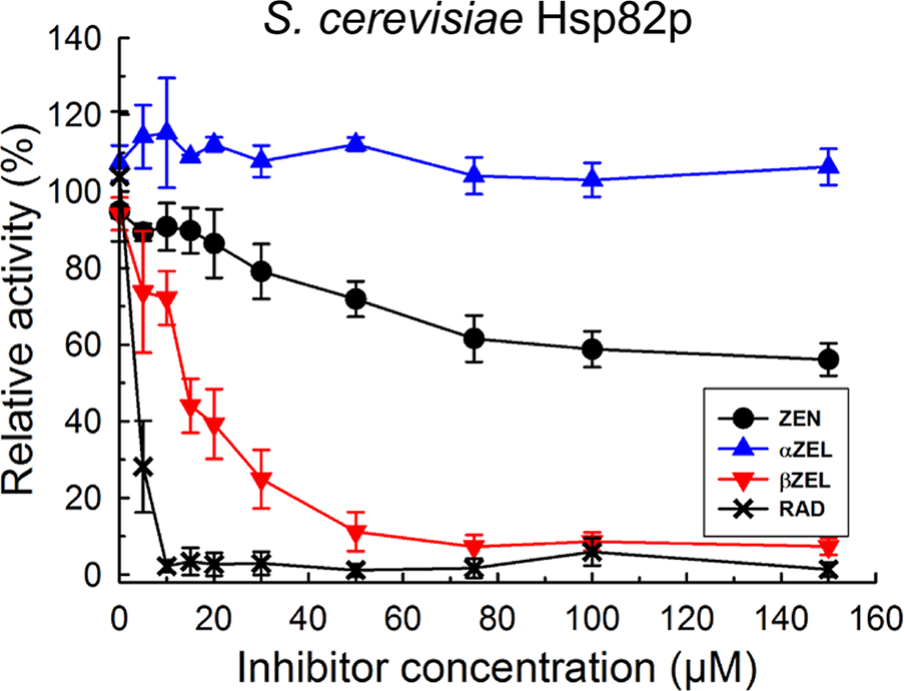
Inhibition of purified 6xHIS-tagged yeast Hsp82p ATPase activity by radicicol (RAD), ZEN, zearalenol-α (αZEL), and zearalenol-β (ßZEL).

According to the model proposed by Yamada and Nishimura (2008), inhibition of HSP90 ATPase activates the heat shock transcription factor and consequently induces various HSP genes. This indicates that ZEN could be an HSP90 ATPase inhibitor. To test this hypothesis, we determined the release of phosphate from ATP by HSP90 using the malachite green colorimetric method as described by Rowlands et al. (2004). We first expressed and purified HSP90 from baker’s yeast, *Sc*Hsp82p, *in E. coli* and observed ATPase inhibition for the positive control RAD, and also for ZEN and its phase I metabolites (**Figure 3**). An IC_50_ value of about 1.5 µM was calculated for RAD, which is close to the value of 0.9 µM reported by Rowlands et al. (2004). Compared to that, ZEN caused a moderate inhibition of *Sc*Hsp82p, with 59 ± 5% (t-test, *p* = 0.0096) of activity at 100 µM. αZEL did not elicit a detectable response, but ßZEL effectively inhibited *Sc*Hsp82p with an IC_50_ of about 15 µM, resulting in an activity reduction to 8.6 ± 2.4% (*p* = 0.0016) at 100 µM.

**Figure 4:**
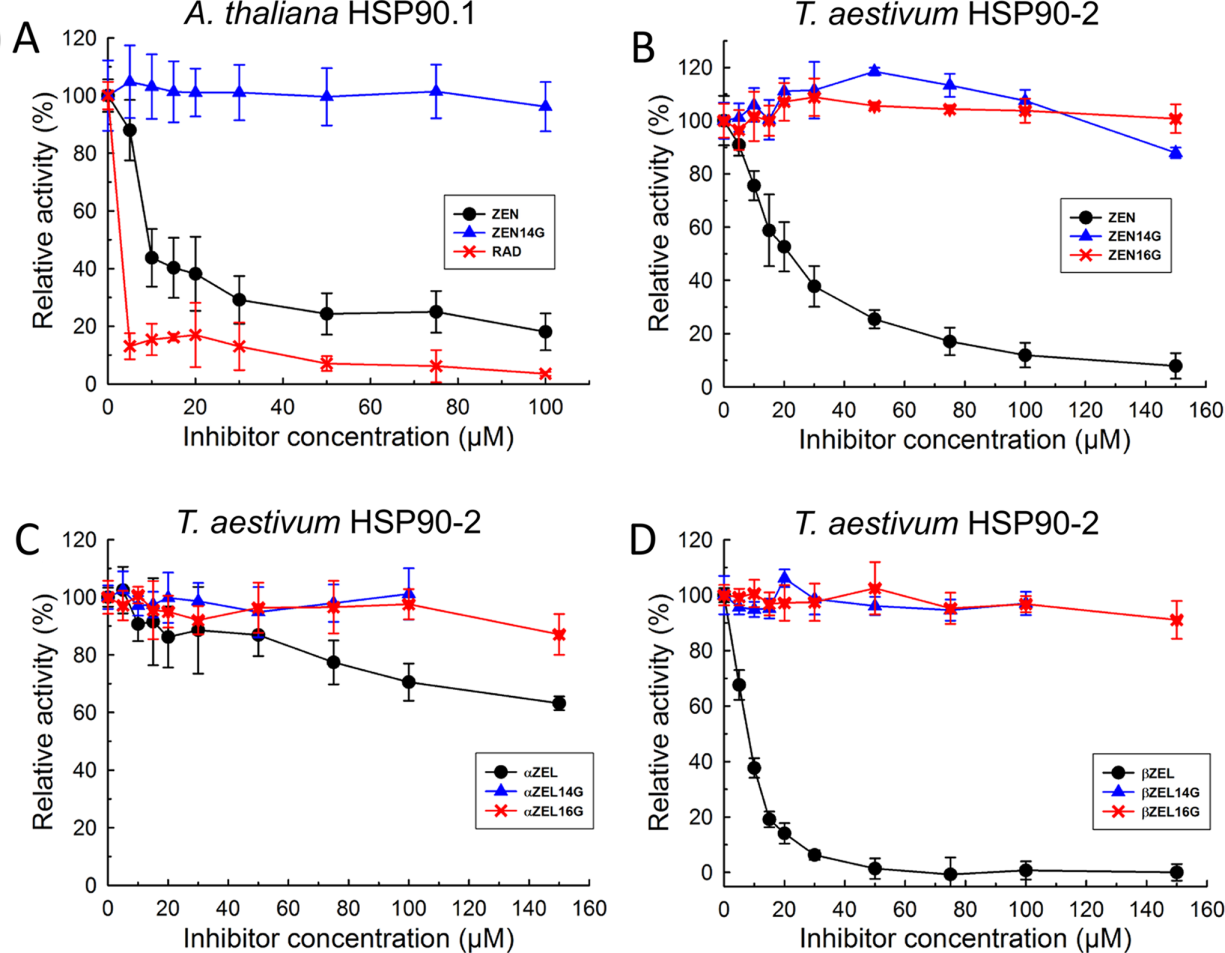
Inhibition of *Arabidopsis thaliana* HSP90.1 **(A)** and *Triticum aestivum* HSP90-2 **(B**–**D)** ATPase activity by RAD, ZEN, zearalenone-14-glucoside (ZEN14G), zearalenone-16-glucoside (ZEN16G), αZEL, α-zearalenol-14-glucoside (αZEL14G), α-zearalenol-16-glucoside (αZEL16G), ßZEL, ß-zearalenol-14-glucoside (ßZEL14G), and ß-zearalenol-16-glucoside (ßZEL16G).

To test whether the yeast model reflects the situation in dicot and monocots plants, *At*HSP90.1 and an HSP90 protein from wheat were expressed and purified. The wheat cDNA previously named “*TaHSP90-2”* corresponds to UniProtKB Q0Q0I7 (Q0Q0I7_WHEAT). This gene belongs to a family of nine highly similar cytosolic HSP90 proteins of wheat, has been named HSP90.3-D1 in a functional study (Wang et al., 2011), and is located on chromosome 5D (= EnsemblPlants Traes_5DL_89CF7F5DE). The results of the ATPase inhibition tests of *At*HSP90.1 (**Figure 4A**) and *Ta*HSP90-2 (**Figure 4B**) indicate that ZEN inhibits these more effectively than the yeast protein (**Figure 3**). At 100 µM ZEN, ATPase activity of *At*HSP90.1 was reduced to 18 ± 6% (*p* = 0.0053) with an IC_50_ of about 10 µM, and that of *Ta*HSP90-2 to 12 ± 5% (p < 0.0001) with an IC_50_ of 20 µM. As observed with *Sc*Hsp82p, ßZEL had a stronger inhibitory effect than ZEN (**Figure 4D**), causing activity reduction to 0.7 ± 3.3% (p > 0.0001) at 100 µM (IC_50_ ≈ 10 µM), while αZEL showed low inhibitory activity (71 ± 6% residual ATPase activity, *p* = 0.0008) at 100 µM (**Figure 4C**).

### Glucosylation Abolishes the Inhibitory Activity of RAD, ZEN, and ZELs on HSP90

**Figure 5:**
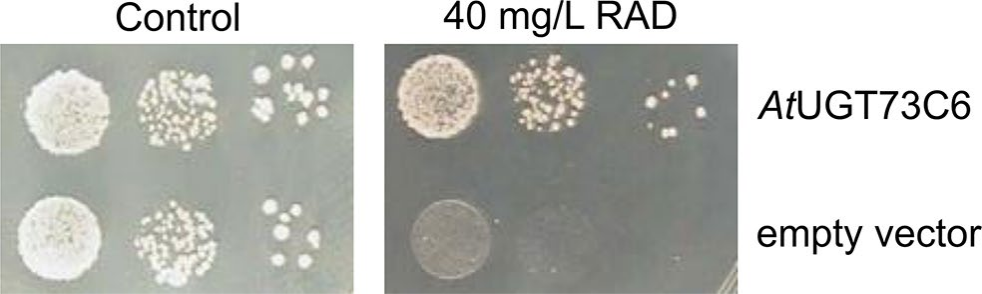
Expression of *Arabidopsis thaliana* UDP-glucosyltransferase *At*UGT73C6 confers radicicol (RAD) resistance to yeast strain YZGA515. Spotting of YZGA515 transformants (three serial 10^−1^ dilutions) expressing either *At*UGT73C6 (upper row) or containing the empty vector. Synthetic complete medium without leucine (SC-LEU) plates without added RAD (left) or with 40 mg/L RAD.

**Figure 6:**
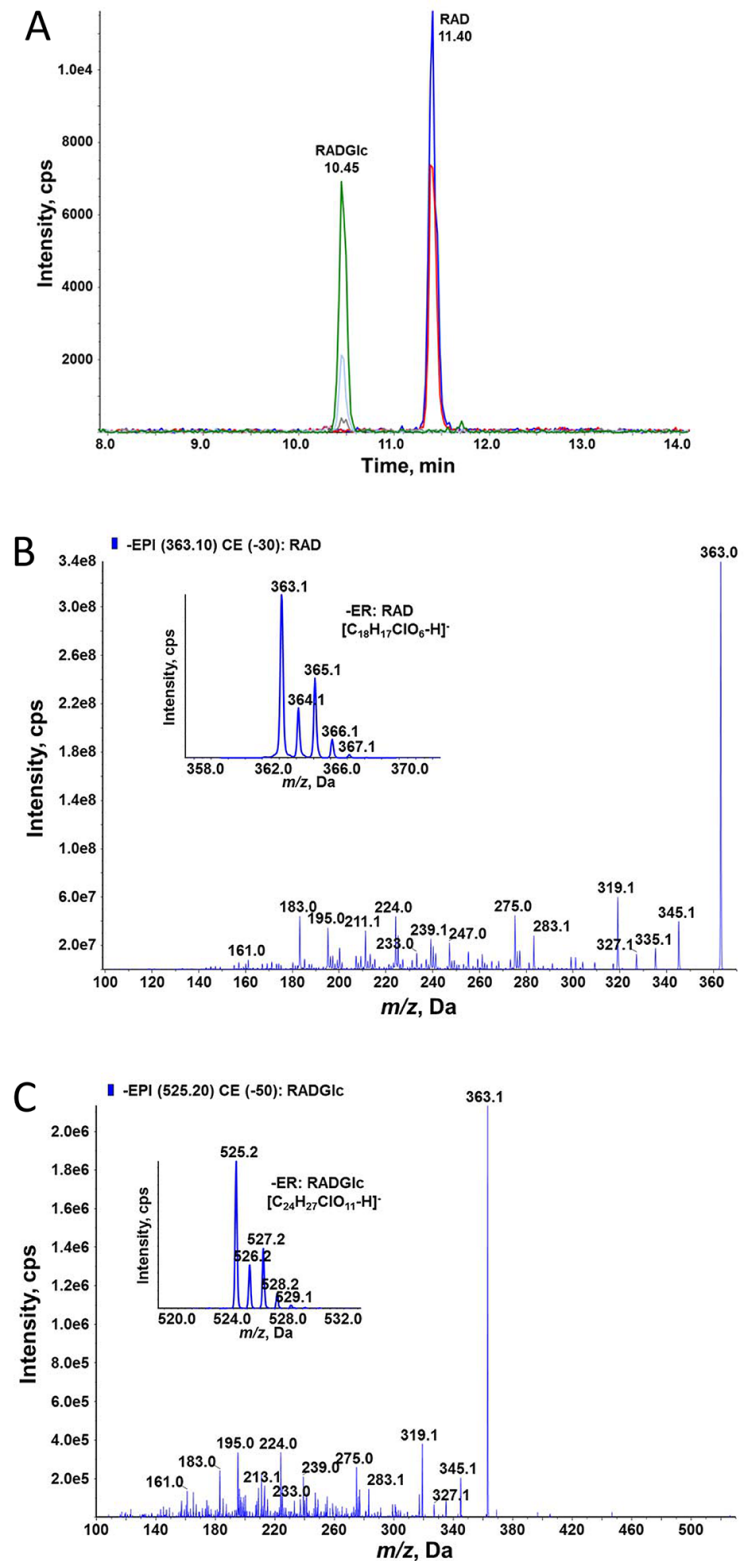
High-performance liquid chromatographic–tandem mass spectrometric (HPLC–MS/MS) determination of RAD and radicicol-glucoside (RADGlc expressing UGT73C6 treated with RAD. **(A)** HPLC–MS/MS analysis in selective reaction monitoring mode of a culture filtrate of yeast. **(B)** Enhanced product ion (EPI) MS/MS spectrum of RAD at 30 eV collision energy; the inlay shows the MS spectrum of the deprotonated ions. **(C)** EPI MS/MS spectrum of RADGlc at 50 eV collision energy; the inlay shows the MS spectrum of the deprotonated ions.

ZEN is known to be converted to glucosides *in planta*; therefore, we tested whether glycosylation of ZEN, α/ßZEL and RAD interferes with HSP90 ATPase inhibition. Previously, we had shown that *At*UGT73C6, when expressed in a multiple-ABC-transporter–deficient yeast strain, leads to the production of ZEN-14-glucoside [old nomenclature ZEN-4-glucoside, Metzler (2011)] after ZEN treatment (Poppenberger et al., 2006). RAD is toxic for this yeast strain, but the expression of the glucosyltransferase *At*UGT73C6 clearly increased RAD resistance (**Figure 5**). We therefore generated RAD-glucoside for *in vitro* tests. RAD was purified from *N. radicicola* and was then used to treat yeast cultures over-expressing *At*UGT73C6. In 50 ml overnight cultures, the formation of a compound consistent with the mass of a RAD-glucoside was detected (**Figure 6**). Since RAD has limited solubility in medium, we produced RAD-glucoside by adding 18 mg RAD to yeast in a small fermenter (see *Materials and Methods*). The formed RAD-glucoside was purified (4.8 mg) and characterized by liquid chromatography coupled to tandem mass spectrometry (LC–MS/MS) and ^1^H-NMR. The MS/MS fragmentation pattern was consistent with a RAD-glucoside (**Figure 6C**). HR-MS measurements yielded the [M-H]^−^ ion of RAD-glucoside with *m/z* of 525.1162, confirming the sum formula of C_24_H_27_ClO_11_ (Δm = −1.36 ppm). Unfortunately, like the parent compound RAD, the glucoside appears unstable, particularly when brought to dryness, so it was not possible to obtain a useful NMR spectrum without a prominent water peak. Therefore, we were unable to determine at which position RAD was glucosylated.

Since *At*UGT73C6 produced exclusively ZEN-14-*O*-glucoside (Poppenberger et al., 2006), we presume RAD was glucosylated at the analogous position. The freshly prepared RAD-glucoside (97% purity) was used to test the activity as an HSP90 inhibitor with recombinant *Sc*Hsp82 protein. We observed no inhibitory activity even at the highest tested concentration of 15 µM, demonstrating that glycosylation of RAD abolishes its activity as an HSP90 ATPase inhibitor. Due to the observed instability of the RAD-glucoside, its stability under assay conditions was evaluated by LC–MS/MS quantification before and after the incubation period. This showed no significant (*p* = 0.21) change in RAD-glucoside concentrations. Likewise, we found all glucosides of ZEN, αZEL, and βZEL stable under identical conditions, with recoveries of > 88%, *p* > 0.05, in each case.

While ZEN was clearly inhibitory to *At*HSP90.1 (**Figure 4A**), ZEN14G was inactive (*p* = 0.75, 100 µM). Likewise, both ZEN14G and ZEN16G did not cause activity reduction of *Ta*HSP90-2 (**Figure 4B**, *p* > 0.1). The results of inhibition tests with the recombinant wheat *Ta*HSP90-2 are displayed in**Figures 4C, D**, showing that the 14- and 16-glucosides of both α- and βZEL were inactive as inhibitors and did not cause significant activity reduction. We therefore conclude that glycosylation is an effective detoxification mechanism with respect to the target HSP90.

## Discussion

In contrast to the mycotoxin and known virulence factor deoxynivalenol (DON), ZEN is formed only late during the infection process. For wheat, cool and rainy weather delaying harvest is a main factor for ZEN contamination that exceeds maximum tolerated levels (Edwards, 2011). Lack of late season moisture typically leads to low ZEN levels even when *Fusarium* head blight severity and DON contamination are high (Kharbikar et al., 2015). Also in corn, where ZEN contamination is more frequent and widespread than in small grain cereals, ZEN accumulates much later than DON during infection of corn cobs (Oldenburg and Ellner, 2015). A recent study employing multiple corn inbred lines revealed clear differences between wild-type and ZEN-deficient *Fusarium* knockout mutants in the employed stalk rot assays with some corn cultivars but not with others (Quesada-Ocampo et al., 2016). The disease symptoms were reduced by about 40%, and also, DON levels were significantly reduced in the stem sections of certain cultivars infected with the ZEN-deficient knockout mutants. In one sweet corn cultivar, the reduction of virulence of the ZEN-deficient mutant was even larger than that observed for a *tri5* mutant unable to produce DON. This study suggests that ZEN may have a role in virulence after all, but that corn genotypes may differ in their sensitivity to ZEN.

In order to improve our understanding of *Fusarium* spp. mycotoxins and their role in plant disease, it is important to identify the mode of action and the molecular targets of these metabolites *in planta*. The transcriptome response of the model plant *A. thaliana* gave a first clue, revealing that *At*HSP90.1 and several other small HSPs were rapidly and highly induced in response to ZEN treatment (Werner, 2005). Together with the structural similarity to RAD, this led to the hypothesis that ZEN targets HSP90. In this work, we could clearly demonstrate that ZEN and, even more so, its phase I metabolite ßZEL are inhibitors of ATPase of monocot and dicot plants and yeast HSP90 proteins. In *A. thaliana* (Berthiller et al., 2006), wheat (Rolli et al., 2018), and various yeasts (Böswald et al., 1995), ZEN is rapidly converted into the stronger HSP90 ATPase inhibitory ßZEL and, to a smaller extent, to the less active αZEL, so it matters which form is preferentially generated by the respective host. Since αZEL has much higher estrogenic activity than ZEN and ßZEL in animals, increasing the conversion of ZEN into αZEL would not be a useful *Fusarium* resistance breeding strategy, if ZEN is indeed a virulence factor. ZEN and ßZEL are much weaker inhibitors than the renowned compound RAD, but the concentrations needed for high-level inhibition *in planta* are clearly within reach (100 µM correspond to 31.8 mg/L). Some strains of *Fusarium* even produce gram amounts of ZEN per kilogram substrate *in vitro*. Naturally infected corn tissue with ZEN contamination levels exceeding 50 mg/kg were reported (di Menna et al., 1997), with local concentrations in infected areas most likely exceeding this by far. The ppm (mg/kg) levels found in *Fusarium*-infected plant material are in agreement with a possible selective advantage to inhibit a very abundant target with a prominent role in disease resistance.

In general, inhibition of the HSP90 ATPase activity leads to ubiquitin-proteasome–dependent degradation of misfolded client proteins. It was shown that treatment with the HSP90 inhibitor geldanamycin reduced the protein levels of the (epitope-tagged) *Pseudomonas* resistance gene products RPM1 and RPS5 in *A. thaliana* (Holt et al., 2005). The hypersensitive response triggered by *Pseudomonas syringae* DC3000 (*avrRpt2*) on *A. thaliana* plants containing the corresponding *RPS2* resistance gene was diminished with 10 μM geldanamycin (Takahashi et al., 2003). Many products of classical disease resistance genes are client proteins of HSP90 and its co-chaperones SGT1 and RAR1 (Shirasu, 2009; Kadota et al., 2010; Kadota and Shirasu, 2012). Downregulation of *HSP90* gene expression through virus-induced gene silencing or pharmacological inhibition of HSP90 activity led to the breakdown of several gene-for-gene resistance interactions (Shirasu, 2009). It has been shown that virus-induced gene silencing of wheat *HSP90* compromises the resistance response to the stripe rust fungus *Puccinia striiformis* f. sp. *tritici*. One example requiring HSP90 is the product of the tomato *I-2* gene conferring resistance to *F. oxysporum* (de la Fuente van Bentem et al., 2005). Since HSP90 is a very prominent target in plant–pathogen interaction, it is surprising that loss of ZEN production in *Fusarium* does not lead to clearly reduced virulence (on the limited number of plants and cultivars tested). The *A. thaliana* microarray data (Werner, 2005) suggest that several detoxification mechanisms, such as ABC transporters, glutathione-S-transferases, and UDP-glucosyltransferases are induced, which could efficiently counteract the *Fusarium* small molecule effector ZEN targeting HSP90. Our finding that the ability to inhibit HSP90 is efficiently blocked by glycosylation suggests that ZEN may be a (nearly) defeated virulence factor, neutralized during coevolution. Nevertheless, this effect could be only partial, and genetic differences in substrate specificity and expression levels of certain relevant glucosyltransferases may exist in the breeding material. Yet, the situation is very complex, as diploid plant genomes contain about 180 UGT genes (Caputi et al., 2012). We have not tested RAD metabolism in plants, but the experiment with yeast expressing the *A. thaliana* UGT73C6 suggests that this compound is also rapidly inactivated into RAD-glucoside *in planta*. This could also be a reason why geldanamycin instead of RAD was used in previous plant studies.

It has been shown that RAD interacts with human and yeast HSP90 with a highly conserved aspartic acid residue (D79 in Hsp82), mediated by water *via* hydrogen bonds (Janin, 2010). The interacting parts of RAD are the hydroxyl groups corresponding to the C-14-OH and C-16-OH in ZEN and the carbonyl oxygen in the lactone. It is therefore not surprising that addition of a bulky glucose molecule to either the C-14 or the C-16 hydroxyl group is sufficient to prevent the interaction with the target. These mentioned structural features are the hallmark of metabolites of the class of resorcylic acid lactones, which together with the similar dihydroxy phenyllactic acid lactones form the group of benzenediol lactones, which are extremely widespread metabolites in (plant pathogenic) fungi, particularly in *Aigialus*, *Cochliobolus*, *Curvularia*, *Fusarium*, *Humicola*, *Lasiodiplodia*, *Penicillium*, and *Pochonia* species. A recent review lists 190 compounds of this class (Shen et al., 2015). Potentially, several of these metabolites could interact with HSP90 and play a role in plant–pathogen interaction. For instance RAD is also produced by the corn pathogen *Colletotrichum graminicola*. Yet, also other proteins with a conserved Bergerat-fold ATP binding site could be targeted. One could also speculate that the diversity of this group of fungal metabolites may be driven by the pressure to escape inactivation by glycosylation or analogous phase II detoxification reactions.

While glycosylation is a detoxification mechanism in plants, it has the consequence that animals and humans consuming contaminating grain are also confronted with glucosides and derived substances [e.g., malonylglucosides, di- and tri-glucosides (Rolli et al., 2018)] which are considered masked mycotoxins, as they are not routinely measured but can be hydrolyzed (Gareis et al., 1990; Binder et al., 2017; Dellafiora et al., 2017; Yang et al., 2018b) back to the parental toxin in the intestinal tract of animals and humans. This may increase the actual mycotoxin burden of populations that, based on measured ZEN, already have a high intake (Rai et al., 2018). Besides its estrogenic activity, ZEN also showed pleiotropic toxic effects in various cell lines and experimental animals when applied in high concentrations (Yang et al., 2018a; Zheng et al., 2018; Cao et al., 2019). It is likely that also, HSP90 of humans and animals is inhibited by ZEN, and the resulting effects on multiple client proteins lead to complex pleiotropic toxicological readouts. HSP90 has not been recognized as a ZEN target in transcriptome studies with mammalian cell lines, which may have the trivial reason that the basal level of HSP90 is already so high that the induced level does not reach a typical cutoff of twofold log2. Also in studies where upregulation of HSP90 (and other HSPs) was noticed (e.g., Hassen et al., 2007), this was attributed to increased oxidative stress triggered by ZEN.

Apart from a role as a virulence factor in plant disease development, ZEN may also have an ecological role in preventing competing fungi from colonizing substrates occupied by ZEN producers and preventing mycoparasitism (Utermark and Karlovsky, 2007) or deterring fungivorous soil organisms. It has been reported that mycoparasitic fungi, e.g., *Gliocladium roseum* (Utermark and Karlovsky, 2007), *Sphaerodes mycoparasitica* (Kim and Vujanovic, 2017), or *Clonostachys rosea* (Kosawang et al., 2014), and also other fungi such as *Rhizopus* species (Brodehl et al., 2014) can counteract ZEN toxicity by formation of ZEN-sulfate, opening the lactone ring, or even by glycosylation.

We could demonstrate here that ZEN targets HSP90 by inhibiting ATPase activity but that this inhibitory activity is effectively antagonized by glycosylation. The taxonomic distribution of ZEN/RAD production in *Fusarium* spp. and other *Hypocreales* suggests that these are ancient metabolites (O’Donnell et al., 2013). It is likely they were neutralized to a large extent by plant glycosylation or similar defense responses in the coevolution between pathogens and plants. This might explain why a virulence function of ZEN is difficult to demonstrate, despite its prominent target with an important role in plant defense.

## Author Contributions

JATA, HM, and GW constructed plasmids and purified recombinant proteins. JATA and HM performed the ATPase activity assay. WS performed the yeast assay and, together with MS, optimized RAD production. MS purified RAD-glucoside, and EV and FB generated and purified the ZEL-16-O-glucosides. EV, FB, and CH analytically characterized the purified glucosides. M-TH, UW, and DM obtained and analyzed A. thaliana HSP90 expression data. GA and RM initially conceived the idea and obtained first preliminary results. GA, RK, M-TH, and FB obtained funding and supervised the experimental work. JATA and HM prepared figures and, together with GA, wrote the draft manuscript, which was commented on, corrected, and finally approved by all co-authors.

## Funding

This work was funded by the Austrian Science Fund (FWF) special research program SFB Fusarium (F3701, F3702, F3706, F3707, F3708, F3711 and F3715).

## Conflict of Interest

The authors declare that the research was conducted in the absence of any commercial or financial relationships that could be construed as a potential conflict of interest.
