# Supplementary material for: Zearalenone and ß-Zearalenol But Not Their Glucosides Inhibit Heat Shock Protein 90 ATPase Activity
Source: Front Pharmacol. 2019 Oct 18;10:1160. doi: 10.3389/fphar.2019.01160 (PMC6813925; doi:10.3389/fphar.2019.01160)
Supplement: Supplementary file 1 [file DataSheet_1.pdf]

Supplementary Figures

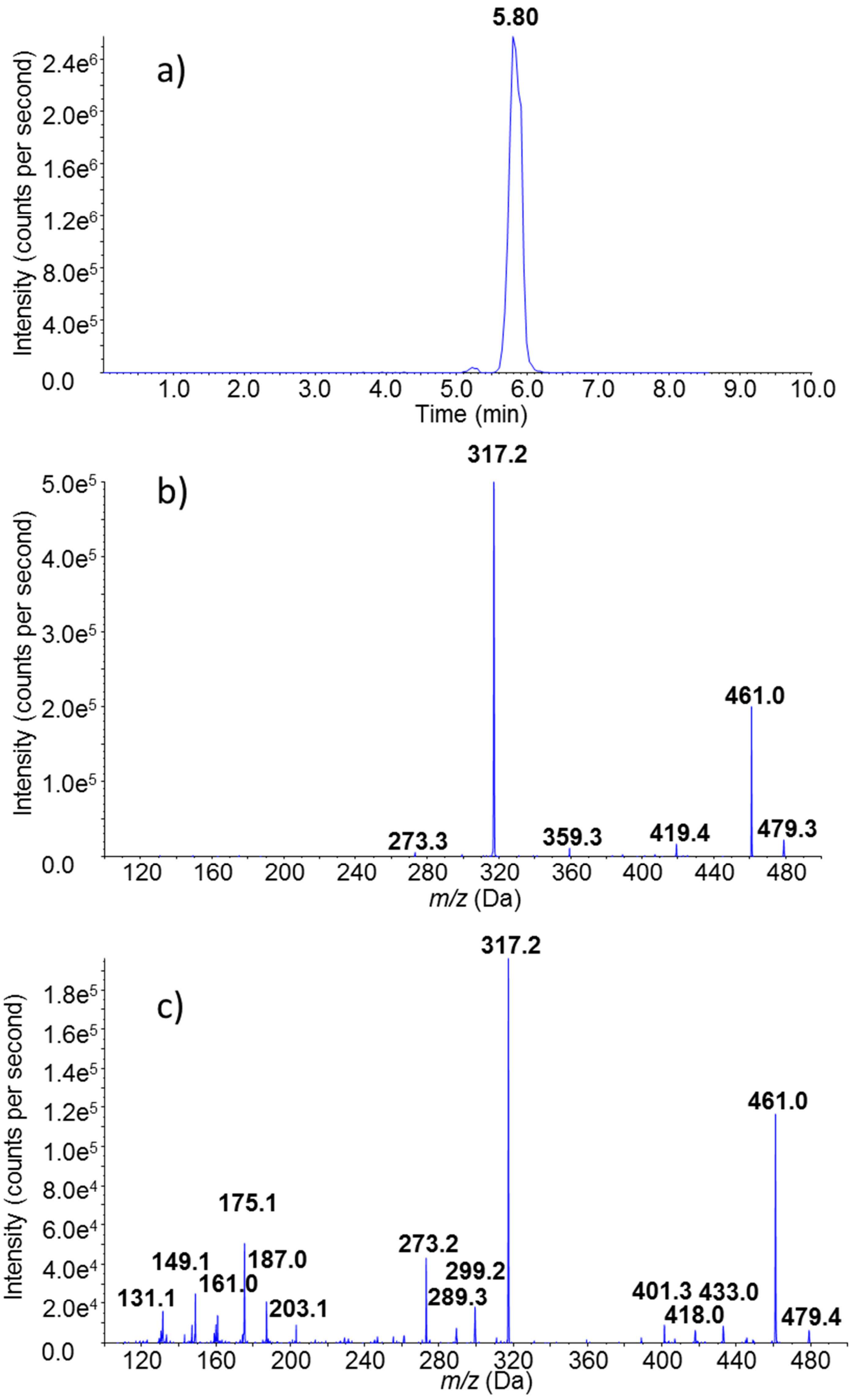

Supplementary Figure S1: Zearalenone-14-*O*-glucoside

a) Extracted ion chromatogram of an enhanced product ion scan (EPI) of *m/z* 479.2 showing *m/z* 316.95 to 317.45
b) EPI scan of *m/z* 479.2 at a collision energy of -30 V
c) EPI scan of *m/z* 479.2 at a collision energy of -50 V

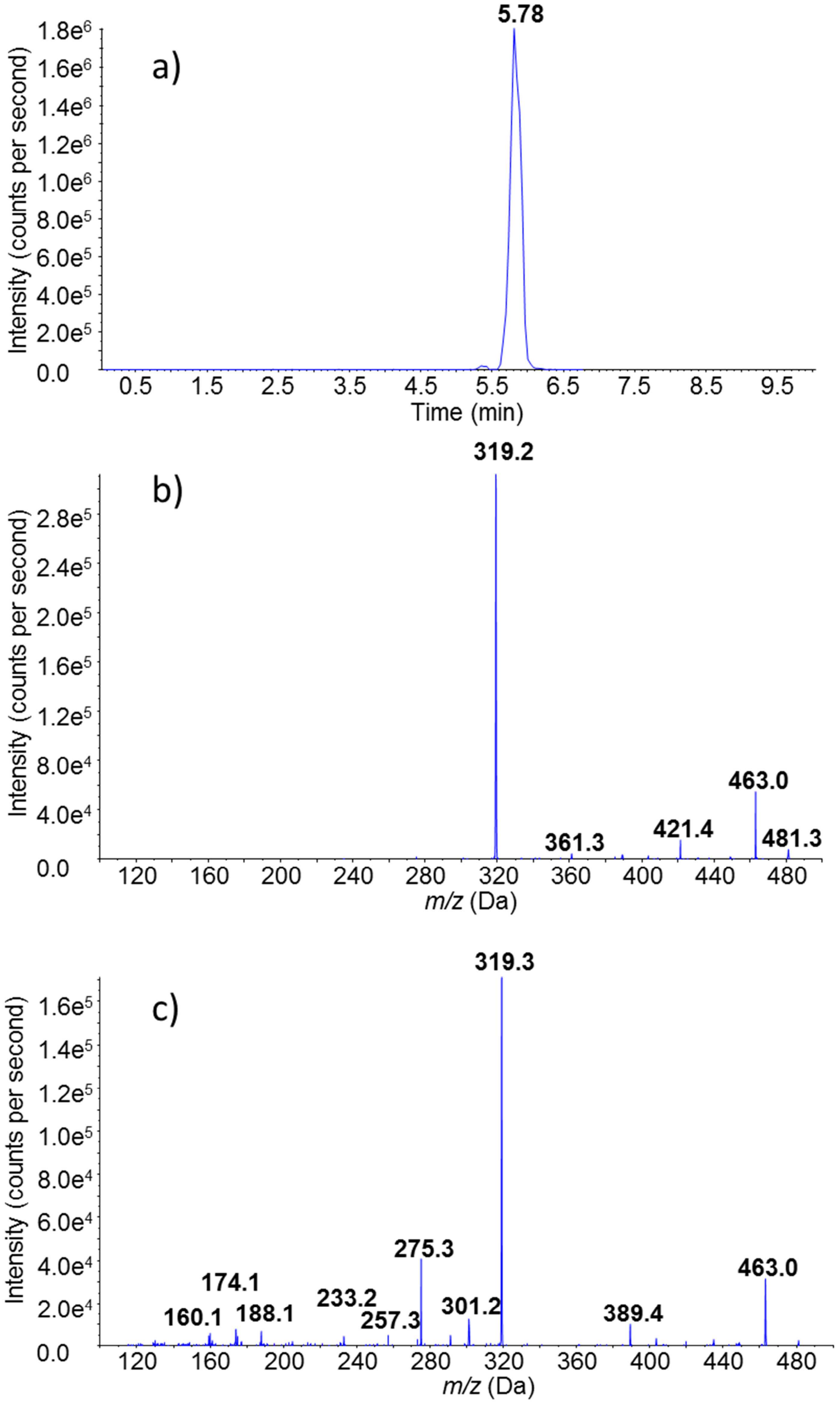

Supplementary Figure S2: α-Zearalenol-14-*O*-glucoside

a) Extracted ion chromatogram of an enhanced product ion scan (EPI) of *m/z* 481.2 showing *m/z* 318.95 to 319.45
b) EPI scan of *m/z* 481.2 at a collision energy of -30 V
c) EPI scan of *m/z* 481.2 at a collision energy of -50 V

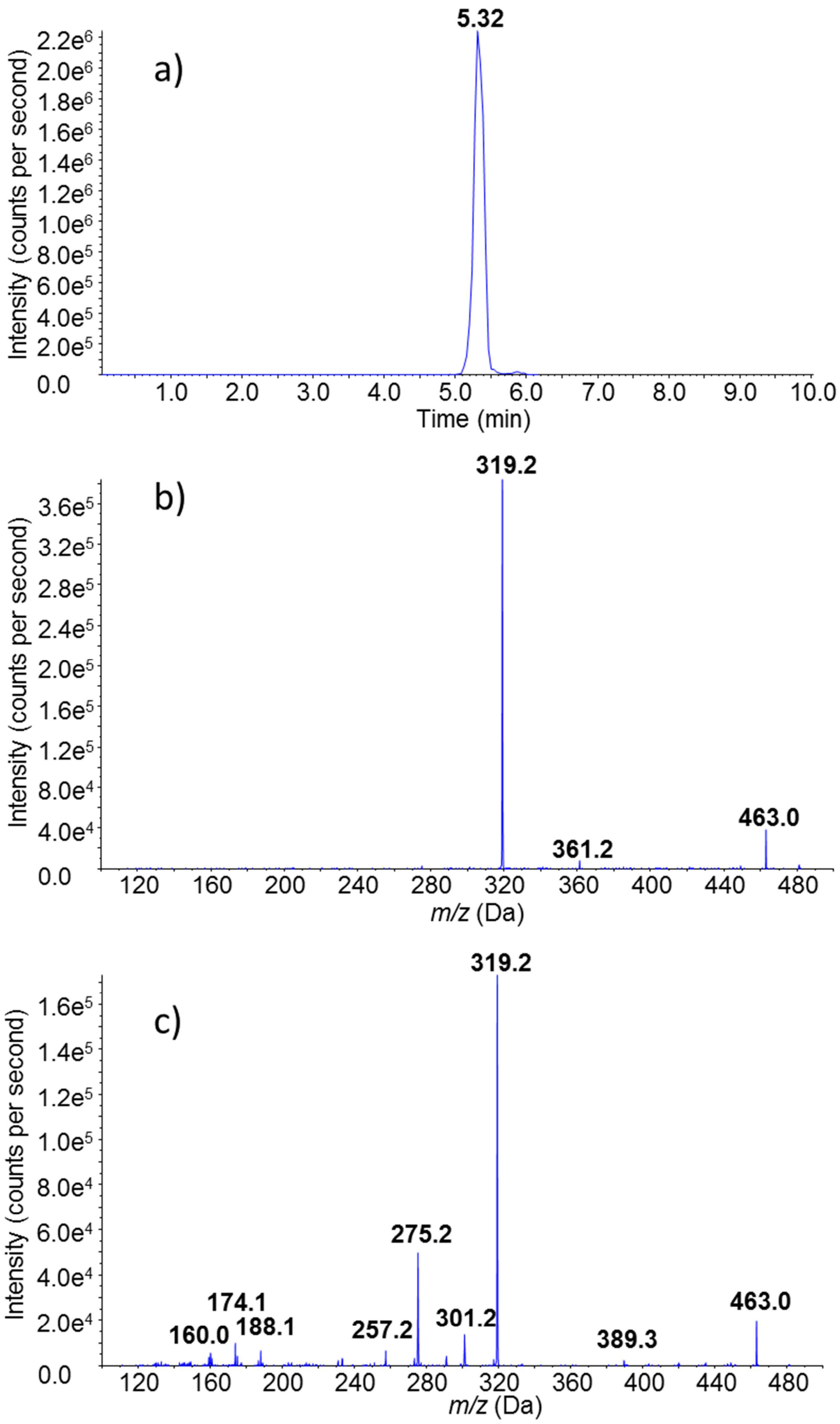

Supplementary Figure S3: β-Zearalenol-14-*O*-glucoside

a) Extracted ion chromatogram of an enhanced product ion scan (EPI) of *m/z* 481.2 showing *m/z* 318.95 to 319.45
b) EPI scan of *m/z* 481.2 at a collision energy of -30 V
c) EPI scan of *m/z* 481.2 at a collision energy of -50 V

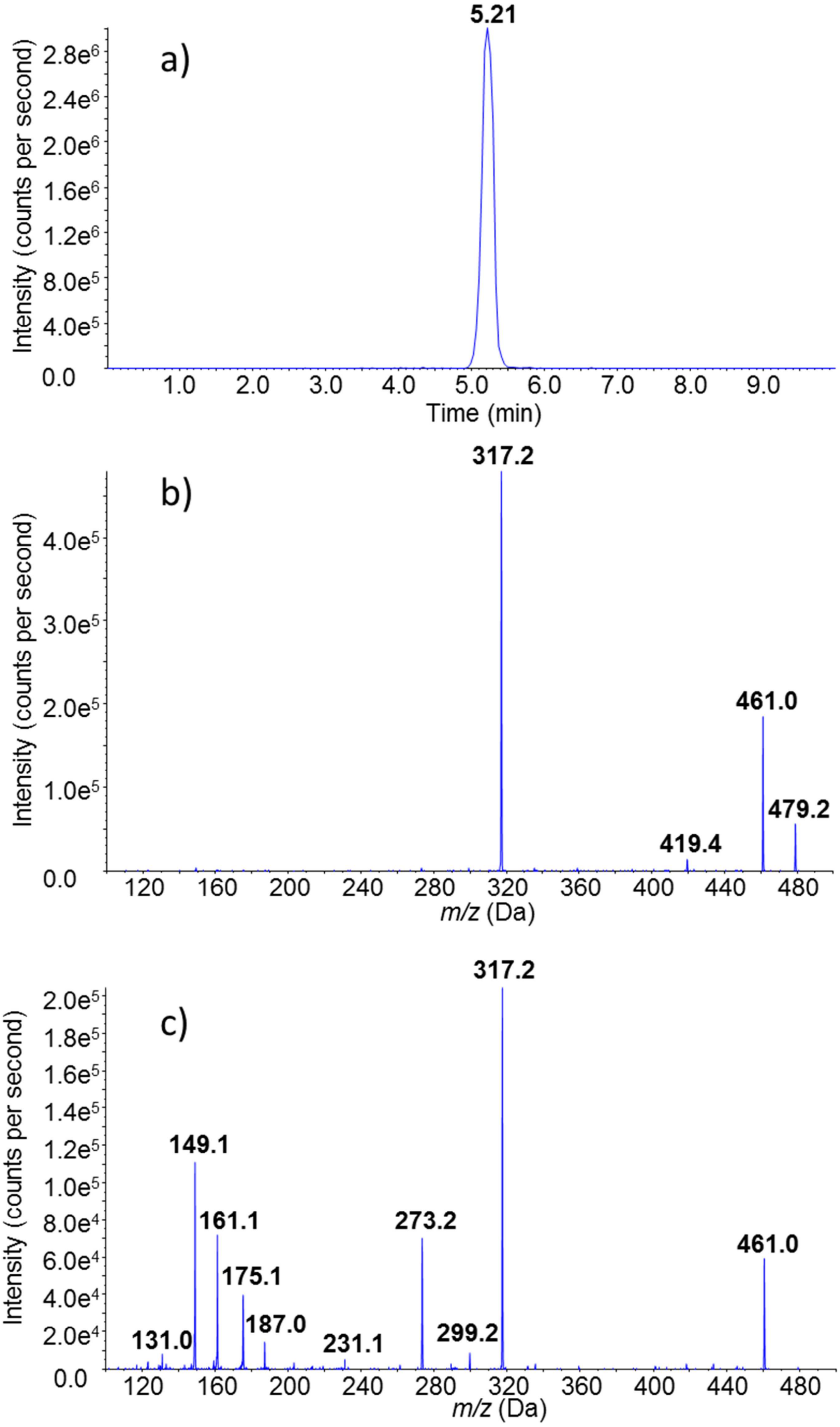

Supplementary Figure S4: Zearalenone-16-*O*-glucoside

a) Extracted ion chromatogram of an enhanced product ion scan (EPI) of *m/z* 479.2 showing *m/z* 316.95 to 317.45
b) EPI scan of *m/z* 479.2 at a collision energy of -30 V
c) EPI scan of *m/z* 479.2 at a collision energy of -50 V

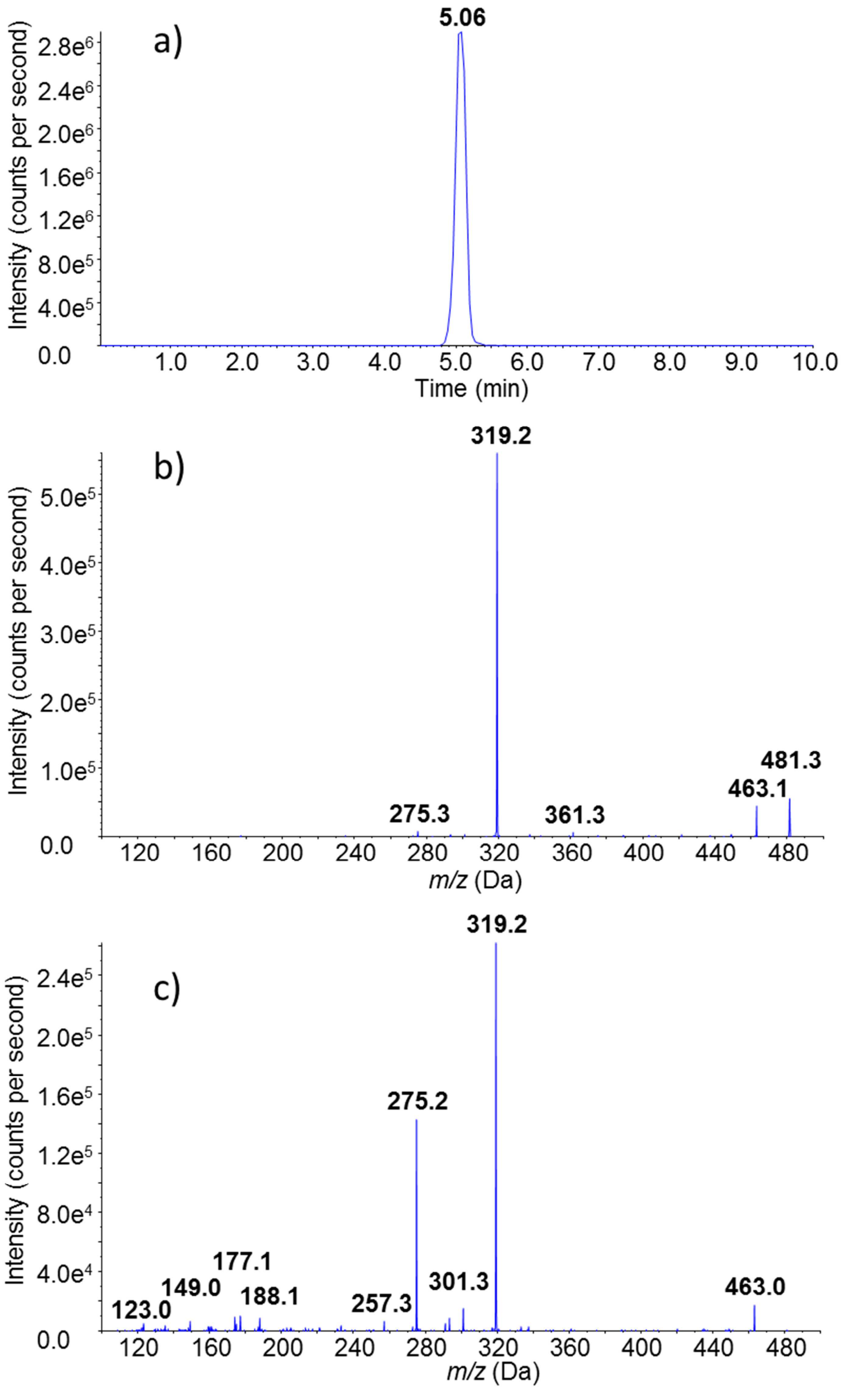

Supplementary Figure S5: α-Zearalenol-16-*O*-glucoside

a) Extracted ion chromatogram of an enhanced product ion scan (EPI) of *m/z* 481.2 showing *m/z* 318.95 to 319.45
b) EPI scan of *m/z* 481.2 at a collision energy of -30 V
c) EPI scan of *m/z* 481.2 at a collision energy of -50 V

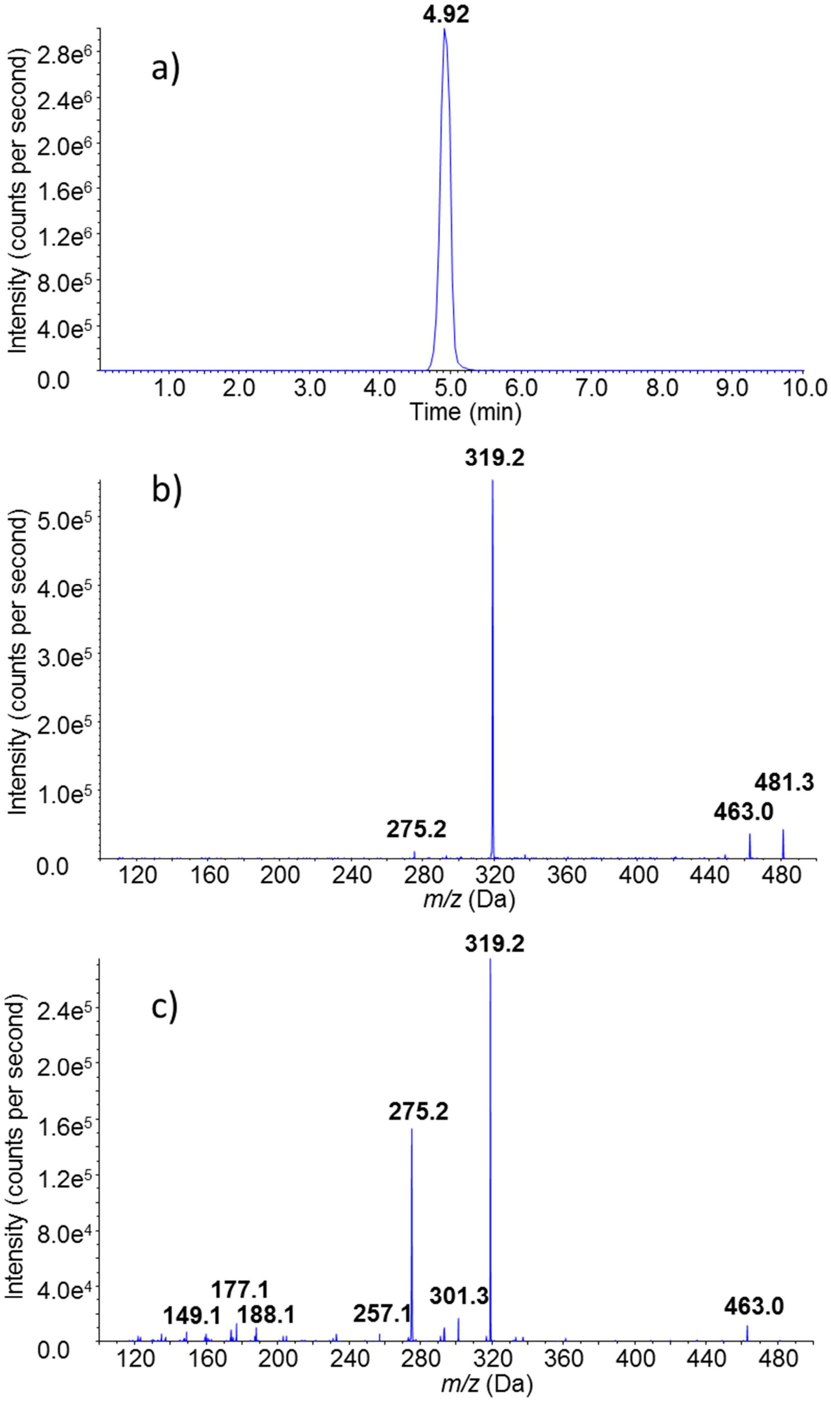

Supplementary Figure S6: β-Zearalenol-16-*O*-glucoside

a) Extracted ion chromatogram of an enhanced product ion scan (EPI) of *m/z* 481.2 showing *m/z* 318.95 to 319.45
b) EPI scan of *m/z* 481.2 at a collision energy of -30 V
c) EPI scan of *m/z* 481.2 at a collision energy of -50 V
